# Editorial: Immunity and Inflammatory Response in Kidney Stone Disease

**DOI:** 10.3389/fimmu.2021.795559

**Published:** 2021-11-01

**Authors:** Visith Thongboonkerd, Takahiro Yasui, Saeed R. Khan

**Affiliations:** ^1^ Medical Proteomics Unit, Office for Research and Development, Faculty of Medicine Siriraj Hospital, Mahidol University, Bangkok, Thailand; ^2^ Department of Nephro-urology, Nagoya City University Graduate School of Medical Sciences, Nagoya, Japan; ^3^ Department of Pathology, Immunology and Laboratory Medicine, College of Medicine, University of Florida, Gainesville, FL, United States

**Keywords:** calcium oxalate, immunomodulation, inflammation, macrophage, microbiota, monocyte, nephrolithiasis, senescence

Kidney stone disease (or nephrolithiasis) is a common urological disorder causing significant morbidity and financial burden in both genders at all ages around the globe (1). Its prevalence is increasing universally at an alarming rate (2–5). Moreover, the stone formation can trigger other renal and vascular disorders such as hypertension, chronic kidney disease and end stage renal disease (6–8). Kidney stones are mineral deposits mostly in the pelvis, free or attached to the renal papillae (9). Calcium oxalate (CaOx) is the main component of approximately 80% of all kidney stones, majority of them being idiopathic (10). Most of the idiopathic CaOx stones develop by attachment to sub-epithelial deposits of calcium phosphate on renal papillary surface, called Randall’s plaques (RPs) (11, 12). Some of the stones form as an overgrowth on crystalline deposits within the terminal collecting ducts of the kidney (13). Both pathogenic mechanisms require periodic urinary supersaturation with respect to CaOx (i.e., hypercalciuria and hyperoxaluria) in association with low levels of its inhibitors (e.g., citrate and other urinary macromolecular inhibitors) (13, 14).

Results of clinical and experimental studies indicate increased expression of genes related to inflammation, immunity and complement activation pathways in renal tissue of experimental animals and stone patients (11). Inflammatory pathways are activated in human renal tissue around the RPs (15). Macrophages appear to be critically involved. M1-related genes are associated with promotion of stone formation, while M2-related genes are related to the stone suppression (16, 17). CaOx crystals induce polarization of M1 macrophages and stimulate inflammatory response in monocytes (18). On the other hand, M2 macrophages can phagocytose and degrade CaOx crystalline fragments (16–18). Macrophage differentiation is also influenced by androgen receptor, which regulates macrophage colony stimulating factor, a cytokine that polarizes monocytes and naïve macrophages into anti-inflammatory macrophages (19).

High oxalate can impact mitochondria of circulating monocytes, leading to altered macrophage polarization (promoting M1 over M2) (20). Immune dysfunction in stone patients may induce oxalate and CaOx-mediated overproduction of reactive oxygen species (ROS) within monocytes, damaging their mitochondria and impairing stone crystal clearance (16–18, 20). Exposure of the naïve bone marrow-derived macrophages to CaOx decreases expression of NAD-dependent protein deacetylase sirtuin-3 and increases proinflammatory mediators (17). In addition to oxidative stress, high oxalate and CaOx crystals can induce inflammatory response through the activation of NLR family pyrin domain containing 3 (NLRP3) inflammasome, which triggers the release of proinflammatory cytokines IL-1B and IL-18 (21, 22). Inactivating NLRP3 may prevent oxalate damage by altering macrophage polarization. Antioxidant treatment of experimentally induced hyperoxaluria in rats also reduces the inflammatory responses and production of the inflammatory mediators (23, 24).

Indeed, inflammation in kidney stone disease can be the upstream (as a pathogenic factor) or downstream event (as a complication). Despite the aforementioned knowledge, the immunity and immune response in kidney stone disease remained unclear (mainly because they were under-investigated) and thus need further elucidations. This Research Topic therefore provides a great opportunity to highlight and promote research in this area. It is a concise collection of most recent basic, preclinical and clinical studies of immune mechanisms and immunomodulation of kidney stone disease.

A systematic review by Taguchi et al. provides an up-to-date knowledge on roles of macrophages in CaOx kidney stone formation. The article summarizes all the findings related to *in vitro*, *ex vivo* and *in vivo* functions of monocytes and all types of macrophages, including non-polarized and polarized ones, in CaOx kidney stone disease.

A research by Ma et al. shows that among C57BL/6N (B6N), 129, B6J and Balb/c mice, high-oxalate diet causes CaOx crystal deposits, increased renal uromodulin expression, renal inflammation and fibrosis only in the B6N mice. Backcrossing the 129 strain with B6N causes CaOx crystal deposits similar to the B6N mice, whereas co-housing study of microbiota adaptation seems to have no effects on CaOx crystal deposits. The authors conclude that genetic background, not microbiota, plays roles in strain-specific hyperoxaluria-induced kidney stone formation.

Another study by Chuenwisad et al. demonstrates that oxalate, CaOx monohydrate and urine from the stone patients, but not the urine from those without stone and untreated control, cause stress-induced premature senescence and telomere shortening in proximal renal tubular cells similar to the positive control, hydrogen peroxide. They also report that the mechanism underlying such senescence induction may be mediated *via* p16 up-regulation and down-regulation of shelterin components.

An animal study by Jin et al. employs a wide variety of techniques to determine the landscape of renal immune cell population in glyoxylate-induced kidney stone model. They demonstrate that short chain fatty acids (SCFAs) prevent glyoxylate-induced kidney stone formation by increasing CX3CR1^+^CD24^−^ macrophage population and decreasing GR1^+^ neutrophil infiltration in the kidney. Moreover, a mechanistic study reveals that such preventive effects of SCFAs is mediated through GPR43, one of the receptors for SCFAs.

A clinical study by Kumar et al. underscores significant impact of diets on the immunity and immune response in kidney stone disease. They show that the high-oxalate diet affects monocyte bioenergetics, mitochondrial complex activity, cytokines/chemokines profile and inflammatory signaling in humans. However, the clinical impact and final outcome of such immunomodulation in kidney stone modulation remain to be elucidated.

Overall, the knowledge offered in these articles is beneficial to build a clearer picture of the immunity and immune response in kidney stone disease. However, more extensive investigations on this Research Topic are still required to further enhance our understanding of the pathogenic mechanisms of kidney stone disease with an ultimate goal to reduce new and recurrent stone formation and to lessen its complications.

## Author Contributions

All the authors edited this Research Topic, wrote and reviewed this article, and approved it for publication.

## Funding

VT is supported by Mahidol University and Thailand Science Research and Innovation (TSRI) (IRN60W0004). TY is supported by The Naito Foundation and JSPS KAKENHI Grant Number JP19H03791.

## Conflict of Interest

The authors declare that the research was conducted in the absence of any commercial or financial relationships that could be construed as a potential conflict of interest.

## Publisher’s Note

All claims expressed in this article are solely those of the authors and do not necessarily represent those of their affiliated organizations, or those of the publisher, the editors and the reviewers. Any product that may be evaluated in this article, or claim that may be made by its manufacturer, is not guaranteed or endorsed by the publisher.

## References

[1] Thongprayoon C Krambeck AE Rule AD. Determining the True Burden of Kidney Stone Disease. Nat Rev Nephrol (2020) 16(12):736–46. doi: 10.1038/s41581-020-0320-7 32753740

[2] Abufaraj M Xu T Cao C Waldhoer T Seitz C D’Andrea D. Prevalence and Trends in Kidney Stone Among Adults in the USA: Analyses of National Health and Nutrition Examination Survey 2007-2018 Data. Eur Urol Focus (2020). doi: 10.1016/j.euf.2020.08.011 32900675

[3] Youssef RF Martin JW Sakhaee K Poindexter J Dianatnejad S Scales CD. Rising Occurrence of Hypocitraturia and Hyperoxaluria Associated With Increasing Prevalence of Stone Disease in Calcium Kidney Stone Formers. Scand J Urol (2020) 54(5):426–30. doi: 10.1080/21681805.2020.1794955 32715836

[4] Wang W Fan J Huang G Li J Zhu X Tian Y. Prevalence of Kidney Stones in Mainland China: A Systematic Review. Sci Rep (2017) 7:41630. doi: 10.1038/srep41630 28139722 PMC5282506

[5] Khan SR Pearle MS Robertson WG Gambaro G Canales BK Doizi S. Kidney Stones. Nat Rev Dis Primers (2016) 2:16008. doi: 10.1038/nrdp.2016.8 27188687 PMC5685519

[6] Bishop K Momah T Ricks J. Nephrolithiasis. Prim Care (2020) 47(4):661–71. doi: 10.1016/j.pop.2020.08.005 33121635

[7] Viljoen A Chaudhry R Bycroft J. Renal Stones. Ann Clin Biochem (2019) 56(1):15–27. doi: 10.1177/0004563218781672 29792045

[8] Ferraro PM Marano R Primiano A Gervasoni J Bargagli M Rovere G. Stone Composition and Vascular Calcifications in Patients With Nephrolithiasis. J Nephrol (2019) 32(4):589–94. doi: 10.1007/s40620-019-00619-w 31165423

[9] Kok DJ Boellaard W Ridwan Y Levchenko VA. Timelines of the "Free-Particle" and "Fixed-Particle" Models of Stone-Formation: Theoretical and Experimental Investigations. Urolithiasis (2017) 45(1):33–41. doi: 10.1007/s00240-016-0946-x 27915394 PMC5250668

[10] O’Kell AL Grant DC Khan SR. Pathogenesis of Calcium Oxalate Urinary Stone Disease: Species Comparison of Humans, Dogs, and Cats. Urolithiasis (2017) 45(4):329–36. doi: 10.1007/s00240-017-0978-x PMC5511574 28361470

[11] Khan SR Canales BK Dominguez-Gutierrez PR. Randall’s Plaque and Calcium Oxalate Stone Formation: Role for Immunity and Inflammation. Nat Rev Nephrol (2021) 17(6):417–33. doi: 10.1038/s41581-020-00392-1 33514941

[12] O’Kell AL Lovett AC Canales BK Gower LB Khan SR. Development of a Two-Stage Model System to Investigate the Mineralization Mechanisms Involved in Idiopathic Stone Formation: Stage 2 *In Vivo* Studies of Stone Growth on Biomimetic Randall’s Plaque. Urolithiasis (2019) 47(4):335–46. doi: 10.1007/s00240-018-1079-1 PMC6417989 30218116

[13] Bird VY Khan SR. How do Stones Form? Is Unification of Theories on Stone Formation Possible? Arch Esp Urol (2017) 70(1):12–27. 28221139 PMC5683182

[14] Sassanarakkit S Peerapen P Thongboonkerd V. Stonemod: A Database for Kidney Stone Modulatory Proteins With Experimental Evidence. Sci Rep (2020) 10(1):15109. doi: 10.1038/s41598-020-71730-3 32934277 PMC7493926

[15] Taguchi K Hamamoto S Okada A Unno R Kamisawa H Naiki T. Genome-Wide Gene Expression Profiling of Randall’s Plaques in Calcium Oxalate Stone Formers. J Am Soc Nephrol (2017) 28(1):333–47. doi: 10.1681/ASN.2015111271 PMC5198277 27297950

[16] Taguchi K Okada A Hamamoto S Unno R Moritoki Y Ando R. M1/m2-Macrophage Phenotypes Regulate Renal Calcium Oxalate Crystal Development. Sci Rep (2016) 6:35167. doi: 10.1038/srep35167 27731368 PMC5059697

[17] Xi J Chen Y Jing J Zhang Y Liang C Hao Z. Sirtuin 3 Suppresses the Formation of Renal Calcium Oxalate Crystals Through Promoting M2 Polarization of Macrophages. J Cell Physiol (2019) 234(7):11463–73. doi: 10.1002/jcp.27803 30588609

[18] Dominguez-Gutierrez PR Kusmartsev S Canales BK Khan SR. Calcium Oxalate Differentiates Human Monocytes Into Inflammatory M1 Macrophages. Front Immunol (2018) 9:1863. doi: 10.3389/fimmu.2018.01863 30186283 PMC6113402

[19] Zhu W Zhao Z Chou F Zuo L Liu T Yeh S. Loss of the Androgen Receptor Suppresses Intrarenal Calcium Oxalate Crystals Deposition *via* Altering Macrophage Recruitment/M2 Polarization With Change of the Mir-185-5p/Csf-1 Signals. Cell Death Dis (2019) 10(4):275. doi: 10.1038/s41419-019-1358-y 30894518 PMC6427030

[20] Patel M Yarlagadda V Adedoyin O Saini V Assimos DG Holmes RP. Oxalate Induces Mitochondrial Dysfunction and Disrupts Redox Homeostasis in a Human Monocyte Derived Cell Line. Redox Biol (2018) 15:207–15. doi: 10.1016/j.redox.2017.12.003 PMC5975227 29272854

[21] Anders HJ Suarez-Alvarez B Grigorescu M Foresto-Neto O Steiger S Desai J. The Macrophage Phenotype and Inflammasome Component Nlrp3 Contributes to Nephrocalcinosis-Related Chronic Kidney Disease Independent From Il-1-Mediated Tissue Injury. Kidney Int (2018) 93(3):656–69. doi: 10.1016/j.kint.2017.09.022 29241624

[22] Singhto N Kanlaya R Nilnumkhum A Thongboonkerd V. Roles of Macrophage Exosomes in Immune Response to Calcium Oxalate Monohydrate Crystals. Front Immunol (2018) 9:316. doi: 10.3389/fimmu.2018.00316 29535716 PMC5835051

[23] Guzel A Yunusoglu S Calapoglu M Candan IA Onaran I Oncu M. Protective Effects of Quercetin on Oxidative Stress-Induced Tubular Epithelial Damage in the Experimental Rat Hyperoxaluria Model. Med (Kaunas) (2021) 57(6):566. doi: 10.3390/medicina57060566 PMC8228054 34204866

[24] Azimi A Eidi A Mortazavi P Rohani AH. Protective Effect of Apigenin on Ethylene Glycol-Induced Urolithiasis *via* Attenuating Oxidative Stress and Inflammatory Parameters in Adult Male Wistar Rats. Life Sci (2021) 279:119641. doi: 10.1016/j.lfs.2021.119641 34043992

